# Weight loss herbal intervention therapy (W-LHIT) a non-appetite suppressing natural product controls weight and lowers cholesterol and glucose levels in a murine model

**DOI:** 10.1186/1472-6882-14-261

**Published:** 2014-07-23

**Authors:** Nan Yang, Danna Chung, Changda Liu, Banghao Liang, Xiu-Min Li

**Affiliations:** Department of Pediatrics, Icahn School of Medicine at Mount Sinai, New York, NY 10029 USA; Department of Preventive Medicine, Icahn School of Medicine at Mount Sinai, New York, NY 10029 USA

**Keywords:** Obesity, C57BL/6 J, High fat diet, W-LHIT natural product, Weight loss, Serum glucose, Cholesterol, PPARγ

## Abstract

**Background:**

The prevalence of obesity is increasing in industrialized countries. Obesity increases the risk of coronary artery disease, stroke, cancer, hypertension, and type-2 diabetes. Unfortunately, conventional obesity drug treatment is often associated with adverse effects. The objective of this study was to evaluate a novel natural formula, Weight loss herbal intervention therapy (W-LHIT), developed from traditional Chinese medicine, for weight control in a high-fat-diet (HFD) induced obesity murine model.

**Methods:**

Two sets of experiments were performed. In experiment 1, 14-week-old C57BL/6 J male mice were fed with HFD for 21days and then separated into 3 weight-matched groups. One group continued on the HFD as obese-controls. Two groups were switched from HFD to normal fat level diet (NFD) and sham or W-LHIT treated. In experiment 2, 25-week-old obese mice, following 2weeks acclimatization, received either W-LHIT or sham treatment while maintained on HFD. In both sets of experiments, NFD fed, age matched normal weight mice served as normal controls. Body weight and food intake were recorded. Epididymal fat pad weight, serum glucose and cholesterol levels, as well as PPARγ and FABP4 gene expression in epididymal fat tissue were analyzed at the end of the experiment.

**Results:**

In experiment 1, W-LHIT treated obese mice lost body weight 12.2 ± 3.8% whereas sham treated mice lost 5.5 ± 2.8% by day 10 after switching from the HFD to the NFD, without reduction of chow consumption. In experiment 2, W-LHIT treated obese mice maintained on the HFD had significantly lower body weight (8 fold less) than the sham treated mice. W-LHIT treatment also reduced epididymal fat pad weight, blood cholesterol and glucose levels versus sham treated mice without reduced chow consumption. In addition, significantly increased PPARγ (peroxisome proliferator activated receptor γ) and FABP4 (fatty acid binding protein 4) gene expression were found in epdidymal fat tissues. Liver and kidney function and hematology testing results of W-LHIT treated mice were within the normal range.

**Conclusions:**

W-LHIT significantly and safely reduced body weight, normalized glucose and cholesterol levels in obese mice, without suppression of appetite, and increased adipocyte PPARγ and FABP4 gene expression.

## Background

In the past 20 years, the worldwide prevalence of obesity has more than doubled. In 2008, more than 1.4 billion adults were overweight [1]. Using the definition of obesity as a body mass index (BMI) greater than or equal to 30, over 200 million of these men and nearly 300 million of these women were defined as obese [1]. The National Health and Nutrition Examination Survey revealed that more than one-third of adult Americans were obese in 2009–2010 [2]. It is projected that obesity prevalence rates for the United States will be more than 40% by 2025 [3]. Obesity is a chronic disease associated with significant morbidity, and has substantial healthcare implications, due to increased risk for diseases including hypertension, diabetes, stroke, inflammation disorders and certain cancers [3, 4]. Overweight and obesity rank as the fifth leading risk for death globally [1]. At least 2.8 million adults die each year worldwide as a result of being overweight or obese [1]. These data demonstrate the major public health challenge of obesity.

The standard treatment for obesity is diet, exercise and behavior modification. More than two-thirds of adults in the United States are either trying to lose weight or to maintain their weight. However, only 20 percent are both eating fewer calories and engaging in at least 150 minutes of physical activity per week [5]. Therefore, lifestyle modification approaches have had low success rates and frequent relapses.

Drug therapy has been utilized as an additional treatment component, although issues of efficacy and safety limit utilization. Current pharmacotherapies include orlistat and lorcaserin, as well as a number of sympathomimetic and antiepileptic drugs. The choice of anti-obesity drugs is often guided by the comorbidities and relative contraindications of the individual patient. Bariatric surgery is an appropriate option for a specific subset of patients, although this may result in serious complications. Due to the possible adverse effects of prescription drugs for obesity and the potential complications of bariatric surgery [6], there is increasing interest in herbal medications for weight loss. Over the counter dietary supplements are widely used by individuals attempting to lose weight, but evidence supporting their efficacy is lacking. As reviewed by Manore in 2012 [7], most dietary supplements only result in less than 2 kilogram (2-3%) weight loss in adults. Certain imported dietary supplements have been found to be adulterated with prescription drugs, including amphetamines, benzodiazepines, and fluoxetine, which has led to an FDA warning against their use [8]. There still remains a significant gap in the area of safe and effective weight control products.

Traditional Chinese medicines (TCM) have a long history of human use in China, Korea, Japan, and other countries for preventing and treating various diseases as well as for maintaining weight control. TCM is also beginning to play an important role in the US, along with other alternative therapies. In the US, TCM is defined as a “Whole Medical System” by the National Institutes of Health (NIH) and the National Center of Complementary and Alternative Medicines (NCCAM) [9]. In 2004, the US FDA provided guidance for investigating botanical drug products, including complex formulas containing several herbs, focusing on safety, efficacy, and consistency. However, there is no FDA approved oral botanical product derived from TCM presently available [9]. Given the reputable safety, efficacy and low cost of TCM for weight control, there is increasing interest in TCM for weight loss. There has been pioneer research in clinical studies [10] and animal models [11] to explore the role of TCM for weight loss. However, experimental evidence based studies of TCM formulations for weight control following oral administration have been limited and underlying mechanisms of TCM for weight control are lacking. In light of our understanding of metabolic mechanisms and principles of TCM formulation, we developed weight loss herbal intervention therapy (W-LHIT), also known as WL-1. Rodent models of high-fat-diet (HFD) induced obesity have been well accepted as important research tools that provide a window into disease pathogenesis and useful preclinical models for investigation of novel interventions for obesity treatment [12]. W-LHIT formula is consist of 6 Chinese herbal medicines, *Ganoderma lucidum* (Ling Zhi), rhizome of *Coptis chinensis* (Huang Lian), *Radix astragali* (Huang Qi), Nelumbo nucifera Gaertn (He Yie), *Chaenomeles speciosa* (Mu Gua), *Fructus Aurantii* (Zhi Qiao)*.* Extract of *Nelumbo nucifera* seed, *Coptis chinensis*, and *Fructus auranti* have been reported with anti-obesity effect in animal models [13–15]. *Radix astragali* and *Ganoderma lucidum* reduced the serum glucose level in diabetic mouse model [16, 17]. *Chaenomeles sinensis* also showed the antihyerplipidemic, and antihyperglycemic effect in diabetic rat. Although, all individual herbal medicines have been investigated in obesity related research, they did not present significant weight loss effect in short period of treatment. The objective of our study is to investigate W-LHIT formula’s effect in a HFD-induced obesity murine model. We evaluated the effect of W-LHIT on body weight, food consumption, and epididymal fat tissue weight. In addition, we observed the effect on serum glucose, cholesterol, and the expression of two genes, PPARγ and FABP4, involved in metabolic pathways.

## Methods

### Mice and reagents

Fourteen-week-old high-fat-diet induced obese and normal chow fed C57BL/6 J mice were purchased from the Jackson Laboratory (Bar Harbor, ME). These mice were maintained under specific pathogen-free conditions according to standard guidelines for the care and use of animals [18]. The study protocol was approved by Institutional Animal Care and Use Committee at Icahn Mount Sinai School of Medicine, New York. HFD chow, prepared by Research Diets, Inc. (New Brunswick, NJ), was composed of 20 kcal% protein, 35 kcal% carbohydrate, and 45 kcal% fat. Normal fat diet (NFD, Purina # 5053, St. Louis, MO), was composed of 23 kcal% protein, 64 kcal% carbohydrate, and 11 kcal% fat.

### W-LHIT preparation and quality control

W-LHIT formulation was developed with dried aqueous extracts of 6 Chinese herbal medicines-*Ganoderma lucidum*, rhizome of *Coptis chinensis*, *Radix astragali*, *Nelumbo nucifera Gaertn*, *Chaenomeles speciosa*, and *Fructus aurantii.* All raw herbs are Chinese origin, which was certified and individually extracted by a good manufacturing product (GMP) facility, Tian Jiang pharmaceutical Co, Ltd, Jiangsu, China. All herbs were extracted with water and then concentrated and dried according to the standard decocting and drying manufacturing process [19]. The dried powder extract was packaged and stored at room temperature in a dry and well-ventilated botanical storage room at Botanical Chemistry Laboratory at Icahn School of Medicine at Mount Sinai. The tests for heavy metal and microbial content were conducted by Tianjian Pharmaceuticoal Ltd. Jiangsu, China and the results met required standards [20–24].

**Figure 1:**
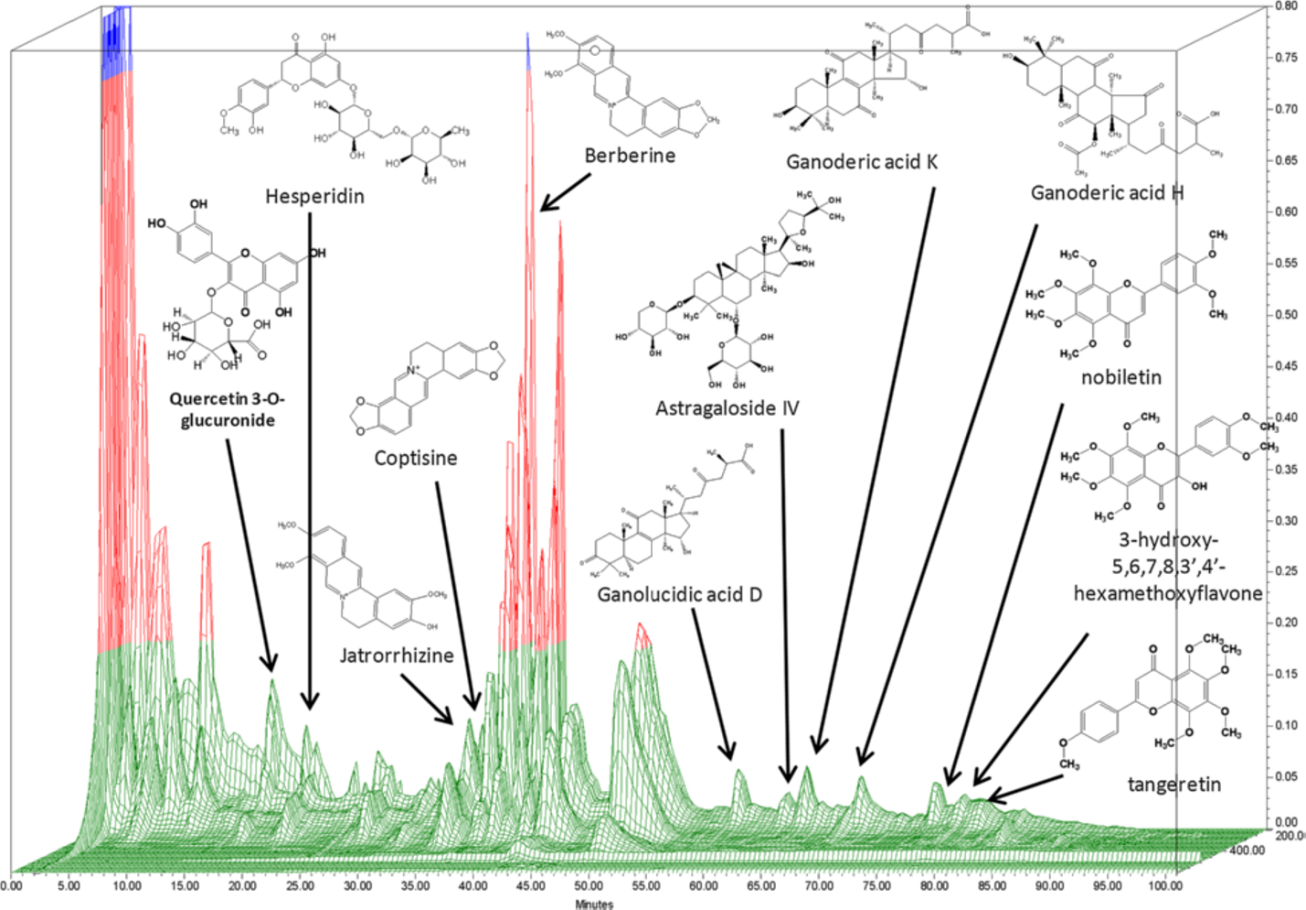
**HPLC fingerprint of W-LHIT.** HPLC conditions: column, Agilent Zorbax SB-C_18_ column (150 × 4.6 mm i.d.; 5 μm particle size); flow rate, 1 mL/min; column temperature, 27°C; mobile phase A, 0.1% formic acid, mobile phase B, acetonitrile. Data were processed using Waters Empower software. Twenty-one major peaks were present in the HPLC fingerprint. Twelve compounds were characterized by LC-MS.

High pressure liquid chromatography (HPLC) fingerprinting is recommended by the FDA as a means of standardization of botanical products. The HPLC fingerprint of W-LHIT was generated using a Waters 2690 HPLC coupled with photodiode array detector (PDA; Waters, Milford, MA). 100 mg of W-LHIT was dissolved into 1 mL of CH_3_CN and 0.1% formic acid mixture (1:1 ratio). The solution was filtered through Whatman 0.45 μm syringe filters (Whatman Inc., Clifton, NJ). 10 μL of filtered solution was injected and analyzed on a ZORBAX SB-C18 (4.6× 150 mm, 5 μm) column (Agilent, Santa Clara, CA). 0.1% aqueous formic acid was used as mobile phase A and CH_3_CN was used as mobile phase B with a constant flow rate of 1.0 mL/min. The gradient was started at 2% B and linearly went up to 25% B within 45 min, then to 35% B within 25 min, to 55% B within 15 min, to 75% B within 10 min, and maintained at 75% B for 5 min. Waters’ Empower software was used for data collection and analysis. A total of 21 major peaks were present in the HPLC fingerprint (Figure 1). Twelve compounds were characterized by Liquid chromatography–mass spectrometry (LC-MS) as quercetin 3-O-glucuronide from *Nelumbo nucifera Gaertn*; hesperidin, nobiletin, tangeretin, and 3-hydroxy-5,6,7,8,3’,4’-hexamethoxyflavone from *Fructus aurantii*; jatrorrhizine, coptisine, and berberine from rhizome of *Coptis chinensis*; astragaloside IV from *Radix astragali*; ganolucidic acid D, ganoderic acid K, and ganoderic acid H from *Ganoderma lucidum*. Their chemical structures and corresponding peaks are shown in Figure 1. Three batches of W-LHIT products were generated. HPLC fingerprints of each individual herbal medicine and comparison of peak intensities of identified compounds were used to monitor the quality of different batches of W-LHIT product. Berberine was used as the key index compound.

### W-LHIT treatment

**Figure 2:**
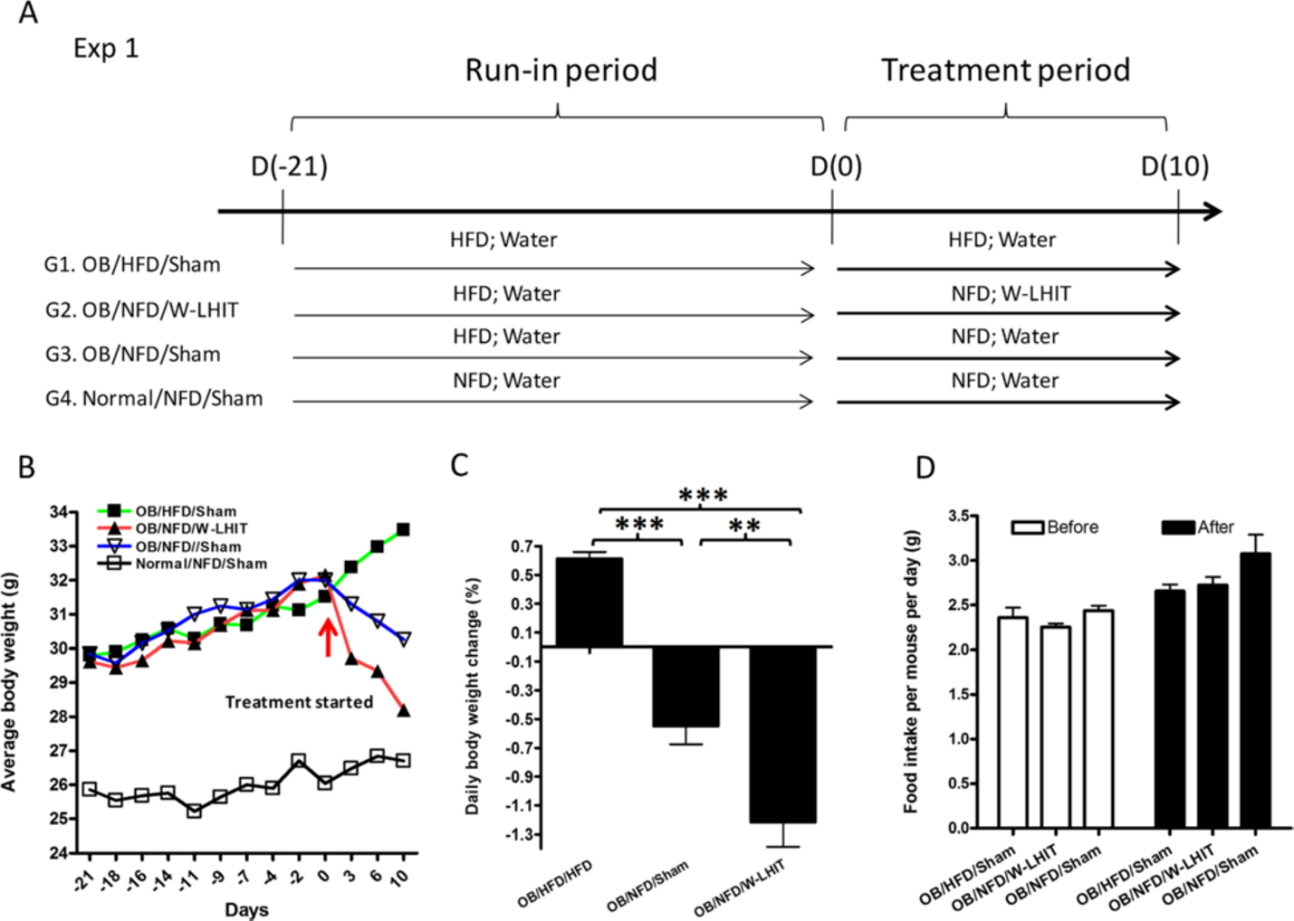
**Effect of W-LHIT on young obese mice bodyweights in experiment 1. A**. Protocols of weight loss experiment 1; **B**. Average body-weight change curve of sham and W-LHIT treated obese mice over time; **C.** Daily body weight change; **D**. Daily food consumption per mouse; **p < 0.01; ***p < 0.001 (n = 5). Data represent two independent experiments.

Each mouse received 84 mg W-LHIT daily, dissolved in 1.0 mL drinking water, and intragastrically (*i.g.*) administered by two separate feedings (0.5 mL per feeding 4 hours apart using a standard mouse feeding needle (VWR, Radnor, PA). The W-LHIT dose was determined by a conversion table of equivalent human to animal dose [25]. We employed two protocols in two sets of experiments to determine the effect W-LHIT on weight control as follows: The first set of experiments was designed to determine the effect of W-LHIT on weight loss as added-on therapy to dietary calorie reduction on young mice. In this set of experiments, three groups of age matched 14 week-old mice (equivalent to human age of 19 years) were first sham treated by *i.g.* administration of water while continuing on the HFD for 3 weeks. This protocol was used to acclimatize mice to *i.g.* administration to prevent potential gavage procedure effect on weight changes (run-in period). Sham treated normal weight mice (G4) fed a NFD served as normal controls. Three weeks later, all mice were weighed. Group 1 obese mice continued on HFD and sham treatment as the obesity control group (OB/HFD/Sham). Both group 2 and 3 obese mice were switched from HFD to NFD, but group 2 mice received W-LHIT (OB/NFD/W-LHIT) whereas group 3 mice received water sham treatment (OB/NFD/Sham). Group 4, the normal weight mice, continued on NFD and water sham treatment to serve as normal controls (Normal/NFD/Sham). Treatment duration was 10 days (Figure 2A).

**Figure 3:**
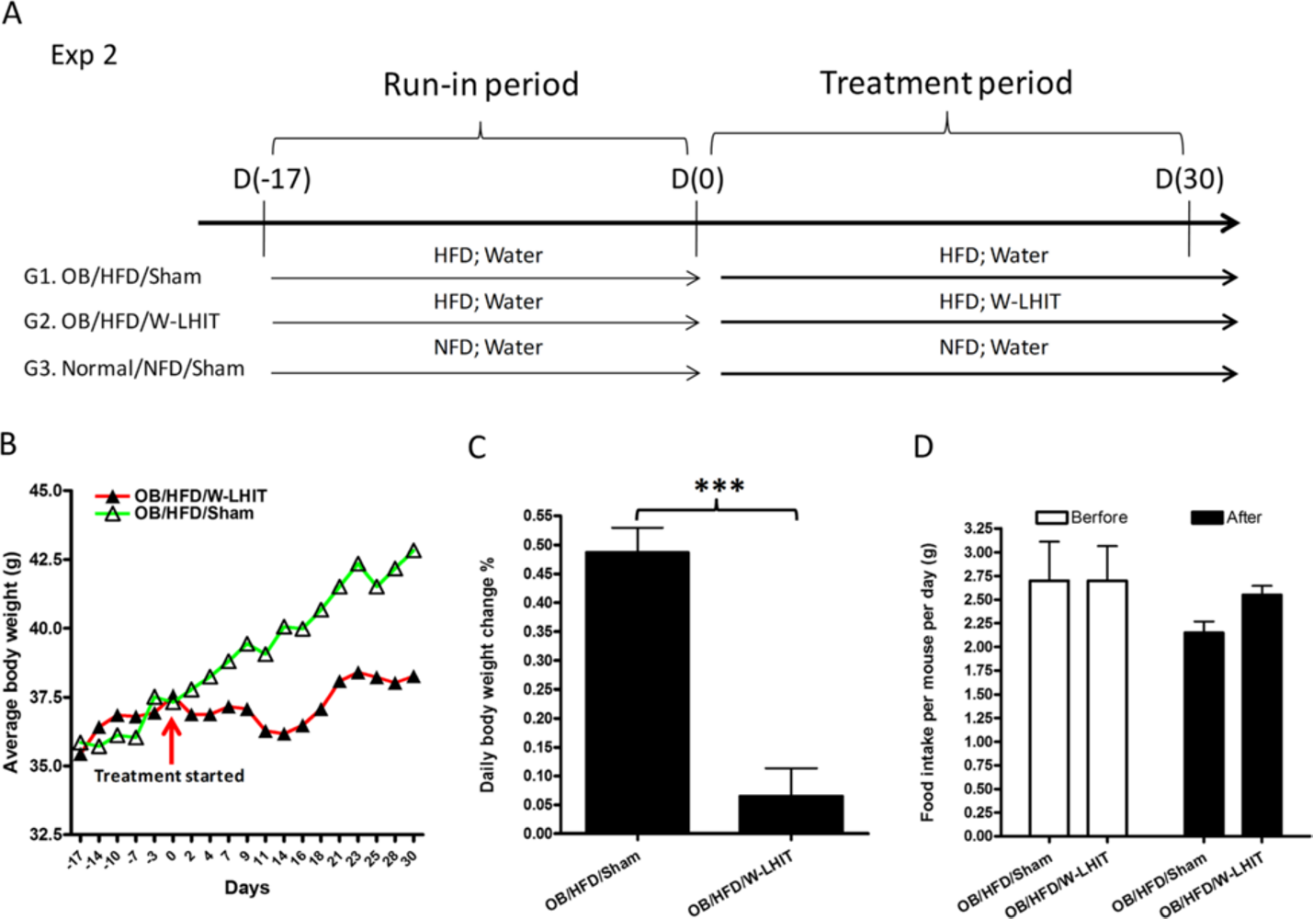
**Effect of W-LHIT on body weights of older obese mice in experiment 2. A**. Protocols of weight loss experiment 2; **B**. Average body-weight change curve of sham and W-LHIT treated obese mice; **C**. Daily body weight change before and after treatment; **D**. Daily food consumption per mouse; ***p < 0.001(n = 5). Data represent two independent experiments.

In experiment 2, to determine the effect of W-LHIT on HFD-induced weight gain in older mice, 14 week-old mice were maintained in an animal facility at Icahn School of Medicine at Mount Sinai for 9 weeks on HFD until 23 weeks old (roughly equivalent to 40 human years). They were then subjected to 2 week acclimatization by *i.g.* water administration. These 25 week-old obese mice were divided into 2 weight matched groups (Figure 3A). Group 1 mice continued on HFD and sham treatment as obese controls (OB/HFD/Sham) while group 2 mice continued on HFD and received W-LHIT treatment (OB/HFD/W-LHIT). Treatment duration was 30 days. Normal weight mice fed with NFD and water sham treatment were used as normal controls (Normal/NFD/Sham).

Body weight and food consumption amounts were recorded three times a week. Body weight gain was calculated by subtracting body weight on the first day from that on the last day of treatment. Daily body weight gain was calculated by dividing body weight gain by the number of treatment days. Chow was weighed three times a week during the period of acclimatization and treatment, and daily food consumption was calculated by dividing total food consumption by the number of days.

### Biochemical analysis

In experiment 2, mice were fasted overnight after 30 days of treatment and submandibular blood samples were collected. Sera were separated and stored at −80°C for further analysis. The mice were sacrificed and tissues were harvested, weighed, and stored at −80°C for further analysis. Serum cholesterol and glucose levels were measured by ALX Laboratories (New York, NY). Since experiment 1 was a preliminary study designed to determine whether the effect of W-LHIT as add-on therapy enhances normal diet intervention weight loss in young obese mice, we did not pursue biochemistry analysis for serum cholesterol and glucose levels in experiment 1.

### RT-PCR

Epididymal fat pads was collected and weighed from mice in experiment 2. Total RNA was extracted from epididymal fat tissue using Trizol reagent (Life Technologies, Grand Island, NY) according to the manufacturer’s instructions. The concentrations of total RNA were measured using optical density (OD) readings (Bio-Rad SmartSpect 3000; Bio-Rad, Hercules, CA). cDNA was then synthesized using ImProm-II™ Reverse Transcriptase Kit (Promega Corporation, Madison, WI) following the manufacturer’s instructions. The real time-PCR reaction was performed by using Maxima™ SYBR Green qPCR Master Mix (2×) kit (Fermentas, Glen Burnie, MD). PCR was started at 95°C for 10 minutes followed by 40 cycles. The temperature profile of each cycle was: 95°C for 15 seconds, 60°C for 30 seconds, and 72°C for 30 seconds. The following primers were used: PPARγ forward: TCGCTGATGCACTGCCTATG; PPARγ reverse: CGAGTGGTCTTCCATCACGG; FABP4 forward: GGATTTGGTCACCATCCGGT; FABP4 reverse: TTCACCTTCCTGTCGTCTGC. Gusb forward: AGTATGGAGCAGACGCAATC; Gusb reverse: CTCTCCGACCACGTATTCTT. All primers were synthesized by Sigma-Aldrich Corporation (St. Louis, MO).

### Safety testing

For acute toxicity analysis, naive mice were fed with 10 times the daily therapeutic dose for mice of W-LHIT and observed for 14 days. In the sub-chronic toxicity assay, naïve mice were fed 5 times their daily therapeutic dose for 14 days. Sham fed mice served as controls (sham). Blood samples were collected after each experiment. Blood urea nitrogen (BUN) and alanine aminotransferase (ALT) measurements for evaluation of kidney and liver functions respectively and complete blood count (CBC) testing were performed by ALX laboratories, NY. Freshly dissected organs from each mouse were collected and fixed in 10% formalin. Organs were then dehydrated and embedded in wax. The sections of different organs were cut and stained with hematoxylin and eosin (H&E). The slides were prepared and evaluated by the Pathology service, Center for Comparative Medicine and Surgery at Icahn School of Medicine at Mount Sinai.

### Statistical analysis

Data were analyzed using SigmaStat 3.5 software (SPSS Inc. Chicago, IL). For data that passed normality testing, differences between groups were analyzed by One Way Analysis of Variance (One way ANOVA) followed by pair wise testing using Bonferroni’s adjustment. For data that appeared skewed (non-normal), differences between groups were analyzed by One Way ANOVA on Ranks followed by all pair wise comparisons. P values ≤0.05 were considered significant.

## Results

### W-LHIT treatment augmented young obese mice weight loss after switching to a reduced calorie diet

In the first set of experiments, we determined the effect of combined interventions on young obese mice by switching from HFD to NFD chow and adding W-LHIT treatment. During the period of 3-week acclimatization, all mice on HFD continued to gain essentially the same amount of body weight (Figure 2B, from days −21 to 0). During the 10-day treatment period, sham treated obese mice remaining on HFD continued to gain weight, 6.1 ± 1.0% by day10. However, obese mice switched to NFD receiving sham treatment (OB/NFD/Sham) lost 2.1 ± 1.4% of body weight by day 3, 3.6 ± 2.5% by day 5, and 5.5 ± 2.8% by day 10. Interestingly, obese mice switched to NFD who also received W-LHIT treatment lost weight more rapidly. Mice in this group lost 7.6 ± 1.5% of their body weight by day 3, 8.7 ± 2.7% by day 6, and 12.2 ± 3.8% by day 10, (Figure 2B). The calculated daily body weight changes over 10-day treatment period showed that body weight increased 0.6 ± 0.1% daily in the OB/HFD/Sham group, but decreased 0.5 ± 0.3% daily in OB/NFD/Sham group, and decreased 1.2 ± 0.4% daily in OB/NFD/W-LHIT group. The daily reduction rate in NFD/W-LHIT mice was 2.4 fold greater than OB/NFD/Sham mice (p < 0.05) (Figure 2C). Daily chow consumption did not differ between W-LHIT treated and Sham treated NFD chow intervention groups (Figure 2D).

### W-LHIT suppressed weight gain in HFD fed older obese mice

In experiment 2, we determined the effect of W-LHIT on 25 week-old male obese C57BL/6 J mice. After the run-in period, mice were divided into 2 groups of equal body weight. Thirty days after initiating treatment, sham treated mice body weight increased by14.6 ± 2.8% while on HFD (OB/HFD/Sham). In contrast, W-LHIT treated obese mice (OB/HFD/W-LHIT) increased body weight by only 1.9 ± 3.3% (Figure 3B). Daily weight gain in the OB/HFD/Sham group was 0.49 ± 0.09% whereas daily weight gain in OB/HFD/W-LHIT group mice was only 0.06 ± 0.10% (Figure 3C, p < 0.001), approximately 8 fold less than sham treated mice. NFD control mice daily weight gain was 0.09 ± 0.06% (data not shown). Food intake did not differ between W-LHIT treated and sham-treated mice while on HFD (Figure 3D).

### W-LHIT reduced epididymal adipose tissue weight in HFD fed older obese mice

**Figure 4:**
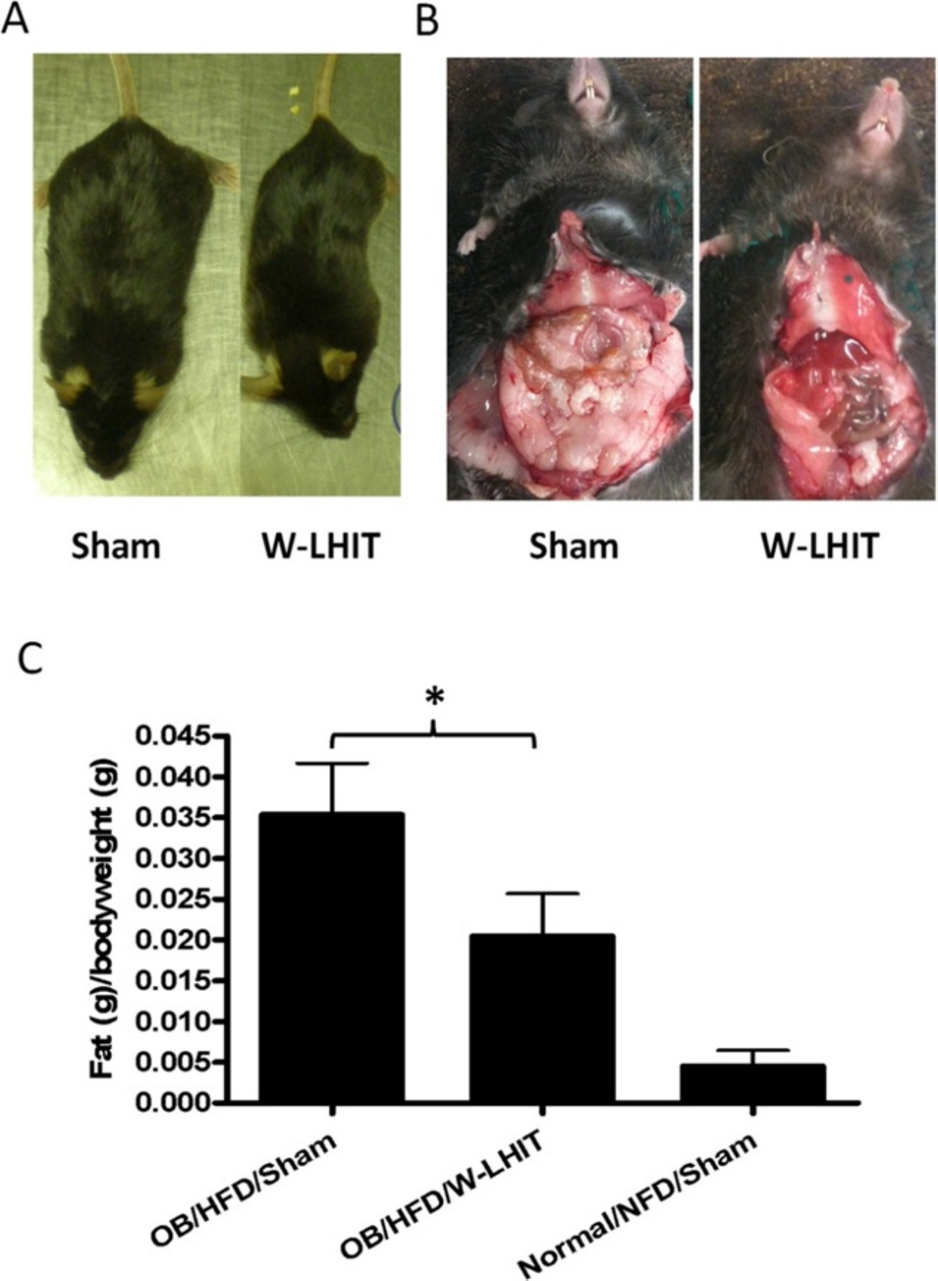
**Effect of W-LHIT on epididymal fat tissue weights of older obese mice in experiment 2. A**. Gross body shape of Sham (left) and W-LHIT treated (right) mice; **B**. Abdominal fat in Sham (left) and W-LHIT treated (right) mice; **C**. Epididymal fat per body weight. Data are expressed as Mean ± S.D. *p < 0.05 (n = 5).

After 30 days on HFD in experiment 2, sham treated mice had increased body size and more visceral fat (Figure 4A). W-LHIT treated mice contained less visceral fat (Figure 4B) and the amount of epididymal adipose tissue was 42% less than that in sham treated mice (Figure 4C, p < 0.05).

### W-LHIT reduced serum cholesterol and glucose levels in HFD fed older mice

**Figure 5:**
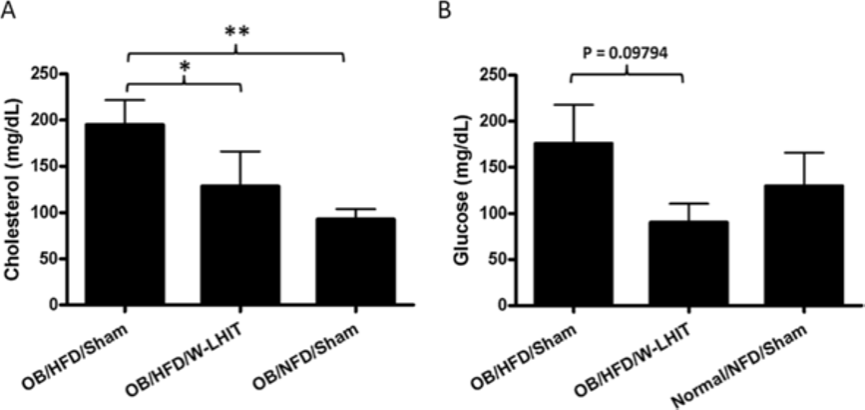
**Effect of W-LHIT on blood cholesterol and glucose levels of older obese mice in experiment 2. A**. Cholesterol levels and **B**. Glucose levels in W-LHIT treated and sham-treated older obese mice and normal controls. Data are expressed as Mean ± S.D. *p < 0.05; **p < 0.01 (n ≥ 4).

In experiment 2, after 30-day treatment, mice were fasted overnight, and blood samples were collected. Serum cholesterol and glucose levels were measured. Mice in OB/HFD/Sham group showed significantly increased serum total cholesterol levels compared with NFD normal controls (195.0 ± 26.8 vs 93 ± 11 mg/dL, p < 0.01 Figure 5A). Cholesterol levels in OB/HFD/W-LHIT group (128.5 ± 37.6 mg/dL) were significantly lower than those of sham treated mice (p < 0.05) and were not statistically different from the NFD normal controls (Figure 5A).

Blood glucose levels in OB/HFD/W-LHIT mice were also lower than that in OB/HFD/Sham mice (90.5 ± 39.6 mg/dL *vs*. 176.0 ± 72.4 mg/dL, p = 0.09), and were not different from NFD normal mice (121.7 ± 62.2 mg/dL, p = 0.35) (Figure 5B).

### W-LHIT increased epididymal fat PPARγ and FABP4 expression in HFD fed older mice

**Figure 6:**
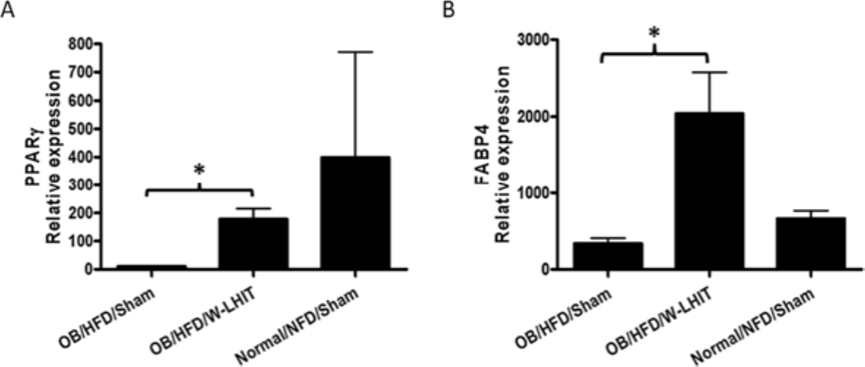
**Real time PCR of epididymal adipose tissues PPAR** **γ** **(A) and FABP4 gene expressions (B) in experiment 2.** Data expressed as Mean ± S.D. *p < 0.05 (n ≥ 4).

Total mRNA was extracted from epididymal fat tissues of sham treated, W-LHIT treated, and normal control mice in experiment 2, and PPARγ and FABP4 mRNA expression were analyzed using real time PCR. W-LHIT treatment significantly increased the expression of PPARγ (p < 0.05, Figure 6A) and FABP4 (p < 0.05, Figure 6B). We also analyzed CPT1, UCP2, and AMPK gene expression, which are related to fat oxidation and metabolism. The results showed that the relative gene expressions of CTP1, UCP2, and AMPK in W-LHIT treated mice also trended upward compared to that in sham treated mice (CTP1, 14.7 ± 12.6 *vs.* 1.14 ± 0.7; UCP2, 83.8 ± 82.6 *vs.* 2.2 ± 3.1; and AMPK, 19.1 ± 15.5 *vs.* 0.2 ± 0.3). However, these increases did not reach statistical significance.

### W-LHIT had a high safety profile

The safety of W-LHIT was tested using 2 protocols. In an acute toxicity assay, mice were fed ten times the W-LHIT treatment dose and observed daily for 14 days. No deaths occurred, and no abnormal behavior or diarrhea was observed (Table 1). In a chronic toxicity assay, mice were fed 5 times the W-LHIT treatment dose for 14 consecutive days. No diarrhea or deaths was observed, and all mice appeared healthy. Mice were then sacrificed and blood samples were obtained.

**Table 1:** **Safety evaluation of W-LHIT treatment**

Treatment			Dose	Time	Death (12 hrs)	Death (24 hrs)	Morbidity percentage		Mortality percentage	
Acute		Water	10X	1 day	0/5	0/5	0		0	
		W-LHIT	10X	1 day	0/5	0/5	0		0	
Chronic		Water	5X	14 days	0/5	0/5	0		0	
		W-LHIT	5X	14 days	0/5	0/5	0		0	
	**BUN (mg/dL)**	**Alanine aminotransferase (U/L)**	**White blood cells (10^3/** **μL)**	**Red blood cells (10^6/** **μL)**	**Hemoglobin (g/dL)**	**Platelets (10^3/** **μL)**	**neutrophils (10^3/** **μL)**	**lymphocytes (10^3/** **μL)**	**eosinophils (10^3/** **μL)**	**Basophils (10^3/** **μL)**
Water	27.0 ± 2.8	27.5 ± 10.6	6.6 ± 3.1	6.6 ± 3.5	13.7 ± 1.0	1155.0 ± 706.8	2920.5 ± 1247.2	3340.0 ± 2248.2	63.8 ± 82.1	0.0 ± 0.0
W-LHIT	21.0 ± 4.4	33.0 ± 5.4	6.0 ± 3.5	8.1 ± 0.6	12.9 ± 0.8	1056.2 ± 526.3	2233.4 ± 1692.3	3499.2 ± 1763.0	52.0 ± 71.3	0.0 ± 0.0
Reference	14-32	16-58	5.4-16.0	6.7-9.71	10.2-16.6	799-1300	1900-3600	8000-18000	0-500	0-400

No morbidity or mortality was observed in acute and chronic W-LHIT treated or normal control mice. Liver and kidney function test and complete blood test results were all in normal range.

Serum ALT and BUN levels were similar to the control group and within the normal range (Table 1). CBC testing was also performed and white blood cell, red blood cell, hemoglobin and platelet levels in the treated group were also within the normal range and similar to the control group (Table 1). These results demonstrated that W-LHIT formula has a high safety profile.

**Figure 7:**
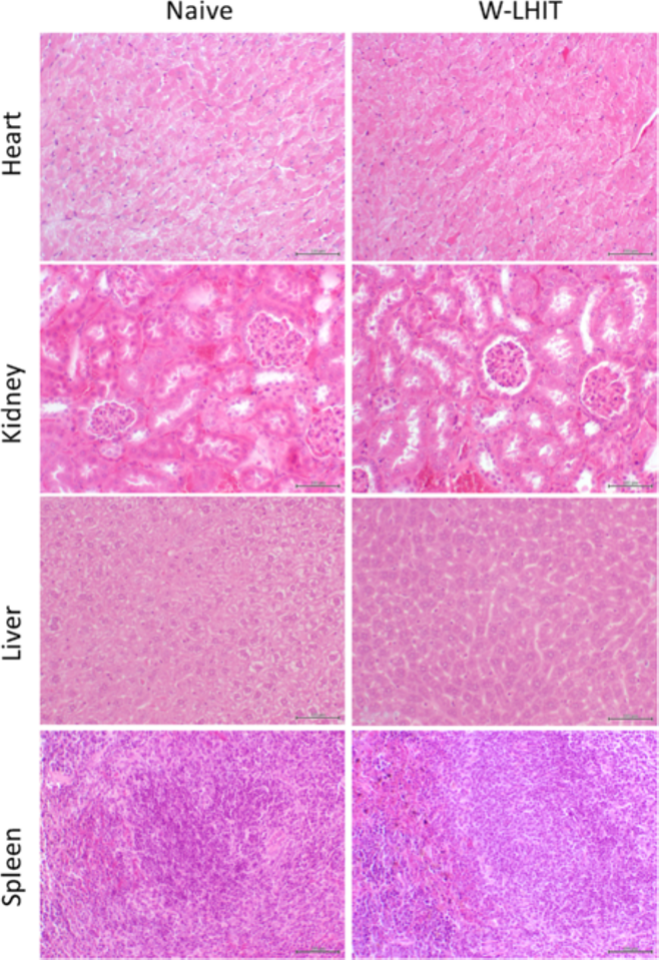
**H&E stained sections of major organs of naïve mice and W-LHIT treated mice.**

Histological sections of the major organs from naïve mice and W-LHIT treated mice were collected and analyzed (Figure 7). Organs examined include heart, kidney, liver, and spleen. No myocardial vacuolation, no necrosis, and no inflammation were noted in hearts. No significant lacy vacuolation and sharply defined vacuolation were observed in liver. No significant difference of renal tubular proteinosis was observed in kidney. Very few tubules contain a homogeneous eosinophilic fluid in kidneys of both naïve and treated mice. No abnormalities were observed in spleens in both naïve and treated mice.

## Discussion

Obesity is a growing concern worldwide, and conventional therapies thus far have proved limited. In this study, we examined the effects of W-LHIT, a natural product developed by our laboratory based on TCM, on HFD-induced obese mice using a previously employed C57BL/6 J murine model fed a 45% kcal HFD [26]. In the first experiment, mice lost body weight when switched from a HFD to a NFD. Additionally, W-LHIT formula treatment accelerated daily weight loss by 250%. This finding suggests that, if the same occurred in humans, W-LHIT as part of a dietary weight loss regimen might help young obese patients lose weight more quickly.

In the US, the prevalence of obesity in individuals over 40 is much higher than that in younger individuals [2] and lifestyle changes including dietary modification have been difficult as a means to stable weight loss for the majority of middle-aged to senior adults. In a second experiment, we employed older mice (middle-aged mice) compared to those in experiment 1 to evaluate if W-LHIT would also suppress HFD induced weight gain without dietary intervention. We found that W-LHIT suppressed daily body weight gain in these mice by approximately 800% as compared to sham treatment. Consistently, W-LHIT also reduced epididymal fat weight. If W-LHIT were to have the same effects in humans, W-LHIT might help limit weight gain in the absence of appetite suppression medications and reduced calorie intake interventions. An additional significant beneficial effect of W-LHIT treatment on HFD fed middle-aged mice was reduction of blood cholesterol and glucose levels. If the same results occur in humans, W-LHIT may be valuable in treating pre-metabolic syndrome and perhaps metabolic syndrome.

Since W-LHIT reduced body weight gain and normalized cholesterol and glucose levels without suppression of appetite in HFD fed middle-aged mice, we hypothesized that W-LHIT may affect signaling pathways involved in cholesterol and glucose metabolism. Fatty-acid-binding protein (FABP4) is predominantly expressed in adipose tissue. Recent research found that adipose tissue in obese individuals exhibited lower FABP4 gene expression than adipose tissue from lean individuals [27, 28]. We found that adipose tissue FABP4 gene expression was significantly increased by W-LHIT treatment. This increased FABP4 expression might have led to the decreased glucose levels and body weight. We also measured gene expression of CPT1, UCP2, which are related to mitochondrial fatty acid oxidation [29, 30], and AMPK, which is important in energy metabolism [31]. All showed a trend of increased expression levels in the W-LHIT treated group. Since W-LHIT did not affect food intake, but did significantly reduce body weight, accompanied by significant reduction of blood cholesterol and glucose levels, we hypothesize that W-LHIT treatment might induce body weight loss through regulating the expression of energy metabolism genes. Previous research showed that activation of PPARγ is mainly involved in regulating lipid metabolism, insulin sensitivity, and glucose homeostasis and its agonist has been used in the treatment of hyperlipidemia and type 2 diabetes [32]. PPARγ reduces cholesterol synthesis and is also important in energy metabolism [33, 34]. In animal study, activation of PPARγ increased insulin sensitivity and reduced the glucose levels in circulation and liver through the regulation of the adipocyte-specific secretory protein, Acrp30 [35]. We found that PPARγ gene expression was significantly increased in epididymal fat tissue from W-LHIT treated mice compared to the sham treated mice. This might, at least in part, explain the decreased glucose and cholesterol serum levels in this study. In addition to increasing insulin sensitivity and reducing glucose levels, PPARγ also has a minor adipogenesis effect [32]. Anti-diabetic Thiazolidinediones drugs (TZDs), PPARγ agonists, have adipogenic effect as unwanted effect. Unlike synthetic TZDs, W-LHIT reduced glucose levels in parallel with the effect of suppression of body weight and serum cholesterol levels. This beneficial effect of W-LHIT may attribute to the combination of herbs in this formula as this formula also enhanced FABP4, CPT1, UCP2 and AMPK to increase metabolism and suppress weight gain. This formula may be advantageous to the single molecule drug that targets on single receptor. Therefore W-LHIT may be a potential as anti-diabetic TZD complementary therapy to reduce TZD adverse effect. At present, it is unknown which active compounds in W-LHIT modulate these gene expressions or molecular mechanisms underlying this gene regulation, so further investigation is required.

The safety of all herbs in W-LHIT is well documented [23, 36]. Notably, this formula does not contain the stimulant Ma Huang (*Ephedra sinica*), which has significant safety concerns when used for weight loss at large doses [7]. We also conducted standard acute and sub-chronic toxicity studies in mice. No mortality or morbidity was observed and no abnormal changes, such as alterations in food and water intake, or diarrhea, were observed. Biochemical analysis data, hematological data and histological investigation results also showed that W-LHIT has a large safety margin.

## Conclusion

In conclusion, we present for the first time evidence of the safety and effectiveness of this Chinese herbal medicine formula, W-LHIT, in high-fat-diet induced obesity in a murine model. W-LHIT treatment augmented weight loss in young obese mice after switching to a reduced calorie diet. It also prevented weight gain in older mice without changing the high fat diet. In addition, beneficial effects were observed on serum cholesterol and glucose levels, perhaps due to the modulation of expression of energy metabolism genes such as PPARγ and FABP4. Further clinical investigation is warranted, as W-LHIT may have potential for promising human obesity treatment.

## Abbreviations

W-LHIT: Weight loss herbal intervention therapy
HFD: High-fat-diet
NFD: Normal fat level diet
PPARγ: Peroxisome proliferator activated receptor γ
FABP4: Fatty acid binding protein 4
BMI: Body mass index
TCM: Traditional Chinese medicines
HPLC: High pressure liquid chromatography
LC-MS: Liquid chromatography–mass spectrometry
BUN: Blood urea nitrogen
ALT: Alanine aminotransferase
CBC: Complete blood count.
